# Naturally-arising amino acid polymorphisms of HIV-1 Nef that differentially modulate downregulation of HLA-A and HLA-B molecules

**DOI:** 10.1186/1742-4690-10-S1-P54

**Published:** 2013-09-19

**Authors:** Macdonald Mahiti, Philip Mwimanzi, Yoko Ogata, Bruce Walker, Zabrina Brumme, Mark Brockman, Takamasa Ueno

**Affiliations:** 1 Center for AIDS Research, Kumamoto University, Kumamoto, Japan; 2 Simon Fraser University, Burnaby BC, Canada; 3 Ragon Institute of MGH, MIT and Harvard University, Boston, MA, USA

## Background

Differential Nef-mediated down-regulation of HLA-Aand HLA-B has been reported in laboratory-adapted Nef strains [1]. Whether naturally-occurring Nef proteins exhibit differential HLA class I (HLA-I) down-regulation activities remains unknown.

## Materials and methods

Plasma HIV RNA-derived Nef clones (one per patient) were isolated from 45 chronically-infected subjects and inserted into the pNL43 proviral vector. Recombinant viruses were prepared and used to infect the HLA-l-deficient cell line 721.221 ectopically expressing either HLA-A*24 or HLA-B*35. Following infection, cell-surface HLA-I expression of virus-infected cells was evaluated by flow cytometry using a pan HLA-I specific antibody [2,3].

## Results

Cell-surface HLA-I expression levels differed following infection with recombinant viruses expressing patient-derived Nef, with median [IQR] expression levels of HLA-A*24 and HLA-B*35 of 38.9 [23.4-76.9] % and 50.7 [39.9-81.9] %, respectively, compared to those of uninfected cells as 100% (*p* <*0.001*)*.* Thus, downregulation of HLA-A by patient-derived Nef clones was significantly more efficient than that of HLA-B, consistent with the previous observations made by laboratory-adapted strains. However, ratios of downregulation activity of HLA-A/HLA-B were median [IQR] 1.25 [0.81-2.37], while that of control strain SF2 was 1.21, indicating a relatively broad range of HLA-A and HLA-B downregulation activities among naturally-isolated Nef clones. Codon-function analysis of HLA-A/HLA-B downregulation ratios identified amino acid polymorphisms at position158 and 202 as being significantly associated (*p* <*0.01*, *q* <*0.2*) with relative abilities to downregulate alleles of HLA-A vs. B loci.

## Conclusions

Despite a broad range of observed function, Nef-mediated ability to downregulate HLA-A exceeded that of HLA-B in 45 Nef clones in chronic infection. We identified for the first time two Nef amino acid polymorphisms at position 158 and 202 that differentially influence HLA-A and HLA-B downregulation, suggesting that they play a role in differential interaction between Nef and allelic polymorphisms of HLA-I cytoplasmic tail.
